# Utilitarian Moral Judgment Exclusively Coheres with Inference from Is to Ought

**DOI:** 10.3389/fpsyg.2017.01042

**Published:** 2017-06-22

**Authors:** Shira Elqayam, Meredith R. Wilkinson, Valerie A. Thompson, David E. Over, Jonathan St. B. T. Evans

**Affiliations:** ^1^Division of Psychology, School of Applied Social Sciences, Faculty of Health and Life Sciences, De Montfort UniversityLeicester, United Kingdom; ^2^Department of Psychology, University of Saskatchewan, SaskatoonSK, Canada; ^3^Department of Psychology, Durham UniversityDurham, United Kingdom; ^4^School of PsychologyUniversity of Plymouth, Plymouth, United Kingdom

**Keywords:** causal inference, defeasibility, deontic introduction, deontic reasoning, deontological moral judgment, is-ought inference, new paradigm, utilitarian moral judgment

## Abstract

Faced with moral choice, people either judge according to pre-existing obligations (*deontological* judgment), or by taking into account the consequences of their actions (*utilitarian* judgment). We propose that the latter coheres with a more general cognitive mechanism – *deontic introduction*, the tendency to infer normative (‘deontic’) conclusions from descriptive premises (is-ought inference). Participants were presented with vignettes that allowed either deontological or utilitarian choice, and asked to draw a range of deontic conclusions, as well as judge the overall moral rightness of each choice separately. We predicted and found a *selective defeasibility* pattern, in which manipulations that suppressed deontic introduction also suppressed utilitarian moral judgment, but had little effect on deontological moral judgment. Thus, deontic introduction coheres with utilitarian moral judgment almost exclusively. We suggest a family of norm-generating informal inferences, in which normative conclusions are drawn from descriptive (although value-laden) premises. This family includes deontic introduction and utilitarian moral judgment as well as other informal inferences. We conclude with a call for greater integration of research in moral judgment and research into deontic reasoning and informal inference.

## Introduction

Amsterdam, 1944. A knock on the door, imperative. At this hour it can only mean one thing; and indeed, it is an SS officer standing at the door. Are all these children yours? He asks. As it happens, they are not: Just before your Jewish neighbor was arrested and sent to a death camp, you had taken her child into your home and promised to treat them like your own. You now face a moral choice: tell the truth, and as a consequence condemn the child to a certain death; or lie, and save their lives (MacIntyre, 1995).

For most of us the question which choice is the moral one is a no-brainer (although we might be tempted to save our own skin). We live in a society where social lies are told as a matter of course. We think little, for example, of complimenting a host on a dish we did not particularly enjoy. Surely telling a lie for the sake of saving lives is not just permissible, but obligatory as well. But for Kant (1998/1785), being consistent in reasoning was an utmost moral obligation, expressed in the categorical imperative, which implies that a reason for an action has to be universal. To have a reason, he argued, is precisely to have a consistent universal principle, and lying to get what one wants cannot be such a principle (Johnson and Cureton, 2016). If all of us lied to try to get what we want, then none of us could get what we want. People have a *deon*, an obligation, as reasoning beings to be consistent; consequential happiness or unhappiness matters little. In the philosophical literature on metaethics, and the parallel psychological literature on moral judgment, a broadly Kantian approach, based on absolute obligations, is therefore dubbed ‘deontological.’

There is an opposite approach, which can be traced back to Hume et al. (2000/1739-1740) and Mill (1971/1861), dubbed ‘consequentialist’ or ‘utilitarian.’ In the utilitarian approach, moral judgment is based on in the consequences of our actions: what is good is what results in the best outcome for the greatest number of people. Thus, lying to the SS officer is a utilitarian choice, because the consequences of lying are better than the consequences of telling the truth. Of course, not all moral dilemmas are as straightforward. In another scenario (Greene et al., 2001), a group is hiding from deadly enemy soldiers. A baby starts crying, threatening to give them all away. The only way to ensure this does not happen is to smother the baby. The utilitarian choice is still the same: take the action that saves the life of many. The nature of the action, however, makes the dilemma much more difficult to resolve.

People can make moral decisions either consequentially or deontologically (or both), and often do: it depends on the nature of the dilemma, as well as individual differences and task demands. A particularly thorny debate revolves around the nature of the psychological processes underpinning each type of moral judgment. Greene (e.g., Greene et al., 2001; Paxton and Greene, 2010; Greene, 2013) famously proposed a dual processing architecture of moral judgment, according to which deontological judgment relies on intuitive, emotion-laden processes, which require little processing effort and are associated with the ventromedial prefrontal cortex; whereas utilitarian judgment relies on analytic, effortful processes, that depend on the dorsolateral prefrontal cortex. This approach ties moral judgment with other dual processing accounts of reasoning, judgment, and decision (Evans, 2008; Kahneman, 2011; Evans and Stanovich, 2013), which make a similar distinction between fast, intuitive processes (a.k.a. Type 1, or System 1), and effortful, resource-hungry processes (a.k.a. Type 2, or System 2). The dual processing approach in general is not without its critics (Kruglanski and Gigerenzer, 2011), and in the psychological literature on moral judgment, where the evidence seems equivocal (e.g., Kahane et al., 2012), the debate has become so central that it is impossible to refer to the deontological/utilitarian distinction without at least mentioning it. However, there is more to moral judgment than dual processing, and in this paper we focus on a different aspect of the distinction between utilitarian and deontological judgment.

## How Do Descriptions Become Norms?

Our research question is this: how do descriptions become norms? Moral dilemmas *describe* situations, yet people readily respond with *normative* judgments. How can people come up with moral normative judgments at all, when the stories of moral dilemmas make no explicit mention of moral terms? To understand the nature of the question, consider another moral dilemma, the staple trolley problem. First proposed by Foot (1967), this is perhaps the most famous moral dilemma of all. Here is Thomson’s (1985, p. 1395) much-cited version:

“Suppose you are the driver of a trolley. The trolley rounds a bend, and there come into view ahead five track workmen, who have been repairing the track. The track goes through a bit of a valley at that point, and the sides are steep, so you must stop the trolley if you are to avoid running the five men down. You step on the brakes, but alas they don’t work. Now you suddenly see a spur of track leading off to the right. You can turn the trolley onto it, and thus save the five men on the straight track ahead. Unfortunately, there is one track workman on that spur of track. He can no more get off the track in time than the five can, so you will kill him if you turn the trolley onto him. Is it morally permissible for you to turn the trolley?”

Here is the rub: the vignette describes a situation, but contains no normative terms as such. There is no mention of terms such as ‘ought,’ ‘permissible,’ ‘must,’ and so on. Yet the question calls for a judgment of permissibility – a normative, or *deontic* judgment. Deontic logic (McNamara, 2010), the logic of norms, of permissions and obligations, is the language we use for moral choice, be it utilitarian or deontological. Deontic logic is a type of modal logic, which uses terms such as ‘obligatory,’ ‘permissible,’ and ‘forbidden.’ Note that *the term ‘deontic’ denotes the logic, whereas ‘deontological’ denotes the type of moral judgment*. *Deontic logic* is the logic of norms and regulations; *deontological judgment* is the moral judgment which relies of rights and duties, i.e., pre-existing norms. Both terms derive from the Greek word *deon*, duty. Deontic logic provides the lexicon and inference rules for *any* moral judgment, be it deontological or utilitarian. [Authors occasionally use ‘deontic’ as short for ‘deontological’ (e.g., Białek and De Neys, 2016), but this practice risks confusion and is best avoided.]

Whether we decide that it is *permissible* to turn the trolley so that the five workmen are spared and the one workman is killed (traditionally considered utilitarian judgment), or that it is *impermissible* to do so (traditionally considered deontological judgment), it is a deontic, normative conclusion that we must draw from descriptive premises. So how can we draw such normative conclusion even though there are no explicit deontic terms in the vignette?

Philosophers call this the ‘is-ought problem,’ or the ‘is-ought fallacy.’ First identified by Hume et al. (2000/1739-1740), the jury is still out on whether such inference from ‘is’ to ‘ought’ is logically valid, and under which circumstances (Hudson, 1969; Schurz, 1997; Pigden, 2010), but this is not our concern here. What concerns us is the psychological mechanism underlying this inference from descriptions to norms. We need no more than task analysis to see that moral judgment requires an inference from ‘is’ to ‘ought,’ because moral judgment requires an inference from a descriptive vignette (‘if I turn the trolley to the side track, I will save the five workmen on the main track’) to a deontic conclusion (‘I must turn the trolley’). Understanding how this inference works can provide some novel insight into the mechanisms of moral judgment.

As a more general observation, we note that, although deontic reasoning is clearly relevant for moral judgment, and although deontic logic provides the computational framework for normative judgments in general and moral judgment specifically, the two research domains generally lead separate lives (although see Bucciarelli et al., 2008, for a rare attempt to connect reasoning and moral judgment; and Holyoak and Powell, 2016, which we discuss in the “General Discussion”). An integrative framework can only benefit theoretical insight in both domains. We see deontic introduction as one such theoretical link, but by no means the only one. This is part of our motivation in the current work.

## Inferring Norms From Descriptions

**FIGURE 1:**
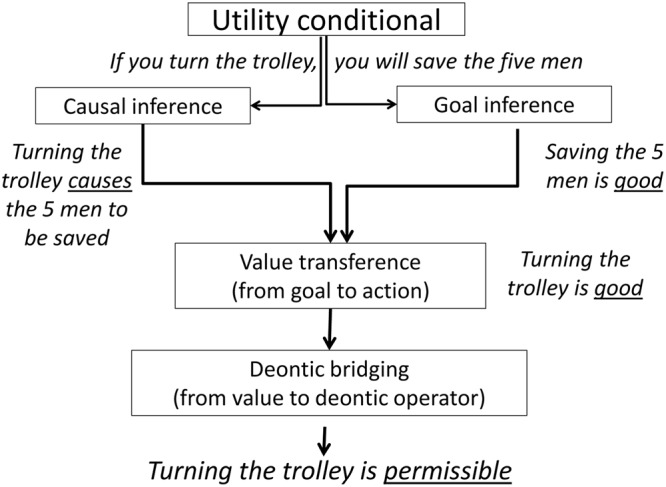
Processing model for deontic introduction. Illustrations from the trolley problem in italics. Adapted from: Elqayam et al. (2015). Copyright 2015 American Psychological Association.

In previous work (Elqayam et al., 2015) we proposed that inference from ‘is’ to ‘ought’ is a pivotal, species-specific process in human cognition, through which humans generate novel norms where no norms existed previously (see also Schmidt et al., 2016; Tworek and Cimpian, 2016). We call this type of inference *deontic introduction*, because the inference *introduces deontic terms* in the conclusion of an argument. Deontic introduction is an informal, pragmatic type of inference, with all this entails: it is sensitive to context, belief and utility; it is enthymematic, defeasible, and probabilistic (Oaksford and Chater, 2007, 2013); and it can be relatively strong or weak (Over et al., 2010; Evans and Over, 2013). Deontic introduction is supported by a largely implicit chain of inference leading from the descriptive premises to the deontic, normative conclusion. The typical context triggering deontic introduction is an action which causally leads to an outcome with desirable or undesirable consequences. For example, given that if Martin uses olive oil his recipe *will* taste better, we readily infer that Martin *should* use olive oil. Under these conditions, people tend to draw deontic conclusions even when there are no deontic terms in the premises. **Figure 1** displays a flowchart of the processing model from Elqayam et al. (2015), with the trolley problem as an illustration.

The input to the inference is an action leading to a desirable (or undesirable) outcome, often expressed in the form of a *utility conditional*; for example, ‘if you turn the trolley, you will save the five men.’ Utility is what we want (positive utility), or do not want (negative utility); a utility conditional is a sentences of the form *if p, then q*, which bears utility (Bonnefon, 2009). Note that this is always presented in context – in this case, the vignette which describes the dilemma.

The next stage in this implicit inference chain includes two interpretative inferences: *goal value inference*, in which people interpret the goal as desirable (or undesirable); and c*ausal inference*, in which people interpret the existence of a causal link between the action and the outcome. In this case, the inferences are ‘saving the five men is *good*’ and ‘turning the trolley will *cause* the five men to be saved,’ respectively. This is followed by *valence transference*, in which psychological value transfers via the causal link from the goal to the action (‘turning the trolley is good’); lastly culminating in *deontic bridging*, where the value transforms into a deontic operator and a new normative rule is created – the ‘ought’ conclusion (‘turning the trolley is permissible,’ or even ‘you ought to turn the trolley’).

The conclusions normally match the utility of the outcome, and the strength of the inference is determined by the strength of the causal link between action and outcome. For example, suppose Sue is packing for a safari holiday abroad but is worried about malaria. Given the premise ‘If Sue takes malaria prevention pills, she will be less in danger of catching malaria,’ participants tend to infer that Sue should take the pills. This conclusion is withdrawn, however, when reasoners are told that the pills are fakes and contain no more than sugar; in other words, when a *disabler* (Cummins et al., 1991; Cummins, 1995) weakens the causal link between action and outcome. Disablers are reasons why no outcome occurs even though the cause is present: in this case, the fake pills, which make it possible that Sue is still unprotected from malaria even though she took the pills. Disablers mean that the cause is no longer sufficient to bring about the effect (Thompson, 1994, 2000). We found (Elqayam et al., 2015) that when the causal link is disrupted, people avoid drawing deontic conclusions. There is a clear parallel to moral judgment here: we know that causality and moral judgment are strongly linked (Sloman et al., 2009; Sloman and Lagnado, 2015; Holyoak and Powell, 2016).

The effect also throws into relief another important feature of deontic introduction: it is, like other types of informal inference, defeasible (Byrne, 1989; Oaksford and Chater, 1998). This means that additional premises make people *suppress* the conclusion: *ceteris paribus*, people infer that Sue should take the pills, but not when they are told that the pills are fake. Deontic introduction can be defeated with added premises that suppress any of the implicit inference steps necessary for chaining ‘is’ to ‘ought.’ Moral judgment, too, is defeasible, as philosophers pointed out (Horty, 1994). Empirical work specifically demonstrating defeasibility is rather sparse, but recent evidence shows that legal arguments, at least, are defeasible (Gazzo Castañeda and Knauff, 2016). Defeasibility also fits with what Holyoak and Powell (2016) called ‘bidirectional inferences’ – the way that people revise their estimates of causes, intentions, moral rules, consequences, and even emotions, to retrofit them to their moral judgments. Such bidirectional inferences draw on the inherent defeasibility of the inferences in moral judgment and extend them. Although much traditional psychological research on reasoning focused on static inference, which is from fixed premises, more attention is now being given to belief revision or updating. This is the result of defeasible dynamic reasoning, in which the premises can become more or less probable or acceptable. Indeed, belief updating and dynamic reasoning are fundamental to the new Bayesian approaches to the study of reasoning (Oaksford and Chater, 2007, 2013). As we acquire new evidence, or more broadly information, we revise the beliefs we rely on for premises and the conclusions we draw from them.

In conclusion, purely on the level of task analysis, there are striking parallels between what we know about moral judgment and what we know about deontic introduction: both call on decision makers to draw deontic (normative) conclusions from descriptions and premises; both are sensitive to the causal structure of the narrative; and both can be suppressed and revised.

## Deontic Introduction and Moral Judgment

In utilitarian as in deontological moral judgment, decision makers need to draw deontic, normative conclusions from descriptive premises. However, there is one obvious difference in the process. Deontological moral judgment, by definition, draws on pre-existing moral norms primed by the vignette; for example, the trolley problem primes something along the lines of *thou shalt not kill*. In contrast, utilitarian judgment, also by definition, requires the decision maker to assess and compare the consequences of each course of action, then draw a tailor-made normative conclusion. What reasoners do when they draw deontic introduction conclusions is in essence the same: they assess the consequences of the action and draw normative conclusions. It is just that moral dilemmas are more complicated than the typical vignettes used in our previous study of deontic introduction, because there are two optional courses of action and the results for each need to be compared. This is not the case, however, for deontological judgment, where decision makers need not calculate the outcome of the action – just the action’s own inherent value, based on that primed moral norm. *Prima facie*, then, it seems that deontic introduction is more closely associated with utilitarian moral judgment than with the deontological sort. In contrast, how strongly the action primes a relevant moral norm would determine the strength of deontological judgment.

Based on this analysis, we propose a *dissociation hypothesis*, in which deontic introduction *exclusively coheres* with utilitarian (but not with deontological) moral judgment. We use the term ‘exclusive coherence’ to refer to a dual pattern, in which deontic introduction *associates* with utilitarian moral judgment, but *dissociates* with deontological moral judgment. We expected exclusive coherence to be articulated as a *selective defeasibility pattern*, in which experimental manipulations known to suppress deontic introduction should suppress utilitarian but not deontological moral judgment. In other words, selective defeasibility is our way to operationalize the dissociation hypothesis. Note that we do not predict a double dissociation, that is, we do not think that priming a deontological norm would affect deontological judgment but not utilitarian judgment. This is because moral norms carry social and symbolic utility, which may come into consequentialist calculations as well. Moreover, our dissociation hypothesis cannot be derived from any other existing theory. For example, Holyoak and Powell’s (2016) deontological coherence framework argues that ‘the inputs to moral judgment processes are intertwined rather than dissociated’ (p. 1193). We discuss the contrasting predictions of this framework in the “General Discussion.”

To test our dissociation hypothesis, we needed to be able to measure utilitarian moral judgment separately from deontological moral judgment. There are well-known issues with the trolley-type paradigm (Cushman and Young, 2011; Fried, 2012; Gold et al., 2014); for the purposes of the current study, our main concern was the forced-choice response paradigm, which confounds action and inaction, and does not allow separate measures of utilitarian and deontological moral judgment. To measure utilitarian and deontological responses independently, we developed a novel experimental paradigm suitable to testing our hypotheses, which we describe in the following section. Two experiments used this paradigm to test our hypotheses.

## Separating Utilitarian From Deontological Measures: The Experimental Paradigm

### The Four Tasks

Recall that our main hypothesis was that the same factors that affect deontic introduction will affect utilitarian moral judgment, but not deontological moral judgment. We therefore needed separate measures for utilitarian and deontological moral judgment. We measured deontic introduction using a utilitarian inference task. We also introduced a parallel deontological inference task. To avoid omission bias (the tendency to prefer inaction to action; see, e.g., Ritov and Baron, 1990; Cushman and Young, 2011) and the action/inaction confound, participants were asked to rate a deontological *action*. This paradigm allowed us to create orthogonal measures for utilitarian and deontological moral judgments, as well as utilitarian and deontological normative inferences.

Each item in each task started with a vignette describing a moral dilemma, with a conflict between a utilitarian outcome and a deontological obligation. Participants were then presented with a utility conditional portraying an action leading to a positive outcome. For example, an anthropologist works with a sect whose members suffer from scurvy because their religion severely limits their fruit and vegetables intake. Persuading them to diversify their diet would reduce the scurvy but offend their religious beliefs. Participants were then presented with a utility conditional, ‘If Ben gets the sect members to eat more fruit and vegetables, he will save them from vitamin C deficiency.’ Each of the four tasks presented the same vignettes and utility conditionals, but with different tasks, depicted in **Table 1**. Participants had to fill in all four tasks, presented in individually randomized blocks. Thus, each individual saw each of the three vignettes four times, each time followed by a different task.

**Table 1:** The four tasks of the experimental paradigm.

Vignette and utility conditional Ben is an anthropologist working with a religious sect in rural America. He gradually realizes that many of his informants suffer from joint pains, bleeding gums, and they bruise easily. He discovers that the sect’s religious beliefs severely limit their fruit and vegetables intake. The result is that many of them suffer from debilitating vitamin C deficiency (scurvy). Ben considers persuading them to eat more fruit and vegetables. *If Ben gets the sect members to eat more fruit and vegetables, he will save them from vitamin C deficiency.*			
		**Utilitarian tasks**	**Deontological tasks**

**Inference tasks**	**Question**	**Utilitarian inference task** Does it then follow: Ben must get the sect members to eat more fruit and vegetables Ben must not get the sect members to eat more fruit and vegetables Ben should get the sect members to eat more fruit and vegetables Ben should not get the sect members to eat more fruit and vegetables Ben may get the sect members to eat more fruit and vegetables Ben need not get the sect members to eat more fruit and vegetables	**Deontological inference task** Does it then follow: Ben must respect the religious beliefs of the sect members Ben must not respect the religious beliefs of the sect members Ben should respect the religious beliefs of the sect members Ben should not respect the religious beliefs of the sect members Ben may respect the religious beliefs of the sect members Ben need not respect the religious beliefs of the sect members
	**Scale**	*1-Definitely does not follow ←→ 7-Definitely follows*	

**Moral rightness tasks**	**Question**	**Utilitarian moral rightness task** Is it morally right or wrong for Ben to get the sect members to eat more fruit and vegetables?	**Deontological moral rightness task** Is it morally right or wrong for Ben to respect the religious beliefs of the sect members?
	**Scale**	*1-Entirely morally wrong ←→ 7-Entirely morally right*	

*Illustrations are taken from the scurvy scenario. All participants received the four tasks.*

#### Utilitarian Inference

To measure deontic introduction we used a *utilitarian inference task* (depicted on the upper-left quadrant of **Table 1**). A variation of the experimental task developed for deontic introduction (Elqayam et al., 2015), it started with the vignette and utility conditional. Participants were then instructed to rate conclusions such as ‘Ben should get the sect members to eat more fruit and vegetables.’ There were six conclusions, each with a different deontic operator (‘must,’ ‘must-not,’ ‘should,’ ‘should-not,’ ‘may,’ ‘need-not’). Participants were instructed to rate how strongly each conclusion followed from the vignettes and the conditional premise, on a fully labeled 1–7 Likert-type scale running from ‘Definitely does not follow’ (1) to ‘Definitely follows’ (7). As far as we are aware, this is a first in terms of testing separately a wide range of deontic operators in moral dilemmas.

Note that the task is *deontic but not deontological:* it is deontic in the sense of deontic logic: it requires participants to infer deontic, normative conclusions (must, should, etc.). However, it is not deontological in the sense of moral judgment, because it does not draw on Kantian ‘hot’ moral judgment.

#### Deontological Inference

To measure how people draw deontological conclusions, we designed a *deontological inference task* (see upper-right quadrant of **Table 1**), in which we asked participants to rate conclusions with the same six deontic operators, but in regard to the deontological course of action. Participants were again presented with the same vignettes and utility conditional as in the utilitarian inference task, but rated different conclusions. For example, in the scurvy scenario, participants were asked to rate conclusions such as ‘Ben should respect the religious beliefs of the sect members,’ using the same scale, from ‘Definitely does not follow’ to ‘Definitely follows.’ This task is both *deontic* and *deontological:* participants are called to draw conclusions containing deontic operators about a course of action underpinned by deontological (Kantian) considerations.

#### Utilitarian Moral Rightness

To measure moral judgment directly, we designed a *utilitarian moral rightness task* (see lower-left quadrant of **Table 1**), in which participants were presented with the same vignettes and utility conditionals as in the other tasks, and instructed to rate whether the utilitarian action was morally right or wrong; e.g., ‘*Is it morally right or wrong for Ben to get the sect members to eat more fruit and vegetables?’* Responses were made on a Likert-type scale from 1 (‘Entirely morally wrong’) to 7 (‘Entirely morally right’).

#### Deontological Moral Rightness

Lastly, the lower-right quadrant of **Table 1** depicts our *deontological moral rightness task*, the deontological equivalent of the utilitarian moral judgment task. Participants were presented with the same vignettes and utility conditionals, and asked to rate the deontological course of action (for example, *‘Is it morally right or wrong for Ben to respect the religious beliefs of the sect members?*’), using the same scale, from ‘Entirely morally wrong’ to ‘Entirely morally right.’

### Defeasibility Manipulations

In two experiments, we capitalized on the defeasible nature of deontic introduction to introduce suppression manipulations. We used three types of suppression as explained below: (a) normative conflict was used in both experiments; (b) goal conflict was used in Experiment 1; and (c) causal suppression was used in Experiment 2. Such suppressions should block deontic introduction by interfering with various links in the implicit inference chain presented in **Figure 1**, which leads from the descriptive premises to the deontic conclusions; and indeed, there is evidence that all three suppression manipulations attenuate deontic introduction conclusions (Elqayam et al., 2015). Thus, they should affect utilitarian, but not deontological judgments. Overall this should result in a consistent pattern of interactions, in which utilitarian, but not deontological dependent measures, vary between levels of the manipulated variable (more on this in the section on hypotheses and predictions). All suppressions were manipulated between participants, such that participants were randomly assigned to one of four (in Experiment 1) or six (in Experiment 2) manipulation cells. Each participant was presented with three vignettes in each of the four tasks.

#### Normative Conflict

In both experiments of the current study, we directly manipulated the salience of the normative conflict between utilitarian outcome and deontological obligation, by either introducing or withholding additional text to the effect that the action violates a relevant professional code of practice; for example, in the scurvy vignette, participants in the high-conflict condition were presented with the following additional text:

However, this would be against their religion. The code of practice of the Anthropological Association requires anthropologists to respect the beliefs of their informants.

We note that in moral dilemmas, conflict is inherent in the very structure of the narrative, and thus impossible to eliminate entirely; it can, however, be either highlighted or downplayed.

Normative conflict interferes with deontic bridging, the transmutation of valence to a deontic conclusion, by introducing a normative rule to the opposite, thus blocking the implicit inference from ‘good’ to ‘should.’ In this case, the inference that Ben should get the sect members to eat more fruit and vegetables is blocked by the opposite norm, namely, that Ben should respect the sect’s religious beliefs, producing the inference that Ben should not get the sect members to eat more fruit and vegetables. In Elqayam et al. (2015), when participants were given additional premises that presented a normative conflict, deontic introduction was suppressed.

Even though the high-conflict manipulation highlights the deontological imperative, we expected deontological responses to be less affected than utilitarian responses, because the cues to trigger deontological responses are already embedded in the vignette. Since deontological judgment is absolute rather than contingent on the context, we did not expect the effects of the vignette and the additional cue to be additive.

#### Goal Conflict

Experiment 1 also manipulated goal conflict, by introducing utility conditionals with the same antecedents but negative instead of positive outcomes. For example, participants in the goal suppression condition received this additional text for the scurvy vignette:

However, the sect members suffer from a rare form of allergy to fruit and vegetables. If Ben gets the sect members to eat more fruit and vegetables, it will drive them into severe, life-threatening allergic reaction.

The theoretical rationale is that goal conflict interferes with valence transference from action to outcome, the penultimate step in the inference chain depicted in **Figure 1**. This is the implicit inference that getting the sect members to eat more fruit and vegetables is good. This inference is blocked, because additional text invites the opposite inference, that getting the sect members to eat more fruit and vegetables is bad. A similar manipulation in Elqayam et al. (2015) significantly attenuated deontic introduction conclusions.

#### Causal suppression

In Experiment 2, we manipulated a different type of suppression, taken from the beginning of the inference chain depicted in **Figure 1** – causality. Experiment 2 manipulated two types of causal suppression, disablers and alternative causes. *Disablers* are reasons why the effect does not happen even when the cause exists; they undermine the sufficiency of the cause to the effect (Cummins et al., 1991; Thompson, 1994, 2000; Cummins, 1995). *Alternative causes* are reasons why the effect can occur even in the absence of the cause; they undermine the necessity of the cause to the effect. In Experiment 2, participants in the control condition were presented with an additional text that emphasized both necessity and sufficiency of the action to the outcome, whereas participants in the suppression conditions were presented with an additional text that included disablers or alternative causes. For example:

***Causal enhancement****: The sect members also avoid any medication or food supplements. The only way to save them from vitamin C deficiency is to get them to eat more fruit and vegetables. Research shows that people with vitamin C deficiency only need to eat fruit and vegetables in order to recover very quickly.*
***Sufficiency suppression (disablers)****: However, it is not enough for Ben to get the sect members to eat more fruit and vegetables. Their staple food is a local type of shellfish, which contains a substance that does not allow the body to absorb vitamin C at all.*
***Necessity suppression (alternative causes):*** *However, it is not necessary for Ben to get the sect members to eat more fruit and vegetables. He can also advise them to eat more beef liver, which contains plenty of vitamin C, and is not against the sect’s beliefs.*

Deontic introduction theory postulates that a crucial part of the implicit inference chain leading to a deontic conclusion is the construction of a causal model (Sloman, 2005) linking the action to the outcome. When participants were given additional information which defeats the causal link, such as disablers or alternative causes, deontic introduction was attenuated (Elqayam et al., 2015). The central role that causality plays in moral judgment is well-established (see Sloman et al., 2009; Sloman and Lagnado, 2015, for reviews), but previous work focused mainly on the type of causal model rather than the strength of the causal link. We are not aware of any previous empirical work that introduced causal defeaters in a moral judgment study.

### Hypotheses and Predictions

Recall that we had a *dissociation hypothesis*, in which deontic introduction exclusively coheres with utilitarian but not with deontological moral judgment; that is, we expect deontic introduction to be associated with utilitarian moral judgment, but dissociated with deontological moral judgment. From this, we derive a *selective defeasibility hypothesis:* Experimental manipulations known to suppress deontic introduction should suppress both utilitarian inference and utilitarian moral rightness ratings, but not deontological inference and deontological moral rightness ratings. From these hypotheses we derive several specific predictions.

We expected two-way interactions between the manipulated variables and moral judgment, such that the moral rightness of the utilitarian action, but not of the deontological action, would be rated lower under test conditions known to suppress deontic introduction: high normative conflict, goal suppression, disabling conditions, or alternative causes. These predictions articulate our selective defeasibility hypothesis, that conditions that suppress deontic introduction suppress utilitarian, but not deontological moral judgment. As the selective defeasibility hypothesis only dictates predictions for each suppression factor, we left open as an exploratory hypothesis the question whether suppression variables interact – that is, whether there would be three-way interactions.

Similarly, we expected three-way interactions between the manipulated variables and inference strength, such that utilitarian but not deontological inference strength would be attenuated under test conditions known to affect deontic introduction (high normative conflict, goal suppression, disabling conditions, or alternative causes): inference strength of conclusions with positive modal operators such as ‘must’ and ‘should’ was predicted to be rated lower, whereas inference strength of conclusions with negative modal operators such as ‘must-not’ and ‘should-not’ was predicted to be rated higher – that is, closer to mid-scale. As was the case for moral judgment, we left open the question of higher-order (in this case, four-way) interaction, because we had no reason to expect the suppression variables to interact or not to interact.

## Experiment 1

In Experiment 1, we manipulated normative conflict and goal conflict. As explained in the previous section, *goal conflict* was manipulated by introducing negative outcomes of the utilitarian action, a manipulation intended to block the inference that the action is good. *Normative conflict* was either kept low, or highlighted by telling participants about a professional code of conduct which creates conflict with the utilitarian action.

If our *dissociation* and *selective defeasibility* hypotheses are correct, both manipulations should affect utilitarian inference and utilitarian moral rightness rating, but not the deontological measures from the right-hand half of **Table 1**: rating of moral rightness of the utilitarian (but not deontological) course of action should be lower in the suppression conditions (i.e., goal suppression, high normative conflict) relative to control, and ratings of utilitarian inference (but not deontological inference) strength should be attenuated, with conclusions with positive operators rated lower, and conclusions with negative operators rated higher (i.e., closer to mid-scale).

### Method

#### Participants

A total of 116 participants took part in an online study using the crowd sourcing platform CrowdFlower and were paid a small sum for their participation. All of the participants had a good record of responding appropriately to quality control questions on other CrowdFlower micro jobs. Responses from 21 participants who were non-native speakers of English or dyslexic, failed to sign the informed consent, or failed a validation question^1^ were excluded from analysis, resulting in a sample of 95, of which 25 were in the low-conflict, no goal suppression condition; 18 were in the low-conflict, goal suppression condition; 28 were in the high-conflict, no goal suppression condition; and 24 were in the high-conflict, goal suppression condition. There were no significant differences between these four groups in sex, age, or level of education. The attrition rate (18%) is comparable to other web studies, and, importantly, there were no significant differences in attrition on the quality control measures between the four experimental test conditions. Participants in the excluded group were significantly younger than the ones in the included group [*M* = 28.1 vs. *M* = 37.2, respectively; *t*(69) = 5.1, *p* < 0.001], but did not significantly differ in their reported level of education.

#### Materials and Procedure

Each participant was presented with the four tasks of the study in individually randomized order: utilitarian inference, deontological inference, utilitarian moral rightness, and deontological moral rightness, as described in the experimental paradigm section. Each task contained one practice item and three individually randomized test items, all presented on the same webpage. The same three items (vignettes and utility conditionals) were presented in all four tasks, and all participants were presented with all items. A full list of items can be found in Appendix A.

#### Design

For moral rightness ratings, we used a 2 × 2 × 2 mixed design, with normative conflict (low normative conflict vs. high normative conflict) and goal conflict (goal suppression vs. no goal suppression) as the between-participants independent variables, and type of moral judgment (utilitarian vs. deontological), as the within-participants independent variable. The dependent variable was rating of the moral rightness of the action, with 7 (‘entirely morally right’) the highest rating and 1 (‘entirely morally wrong’) the lowest.

For the inference tasks, we used a 2 × 2 × 2 × 6 mixed design, with normative conflict (low normative conflict vs. high normative conflict) and goal conflict (goal suppression vs. no goal suppression) as the between-participants independent variables, and type of inference (utilitarian vs. deontological) and modal operator (‘must,’ ‘must-not,’ ‘should,’ ‘should-not,’ ‘may,’ ‘need-not,’ ‘going-to,’ ‘not-going-to’), as the within-participants independent variables. The dependent variable was rating of how strongly the conclusion follows, with 7 (‘Definitely follows’) the highest rating and 1 (‘Definitely does not follow’) the lowest. Thus, higher ratings indicate that the inferences are judged to be stronger.

### Results and Discussion

Here and throughout this report, we report mean scores computed across test items. Where sphericity was violated, we report Greenhouse-Geisser adjusted degrees of freedom. For multiple comparisons we used the Bonferroni correction. We do not report main effects and lower-order interactions where these were modified by higher-order interactions.

#### Moral Rightness

**FIGURE 2:**
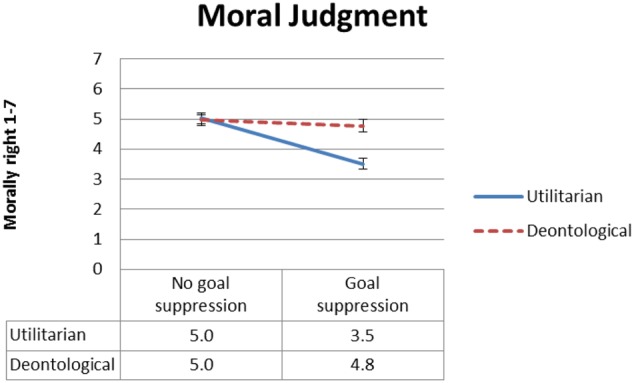
Experiment 1: Mean moral rightness ratings, from 1 (entirely morally wrong) to 7 (entirely morally right), as a function of type of judgment and goal conflict condition. Error bars represent 1 SE. The difference between the goal suppression condition and the no-suppression condition is significant for utilitarian (solid blue line) but not for deontological (dashed red line) judgment.

**FIGURE 3:**
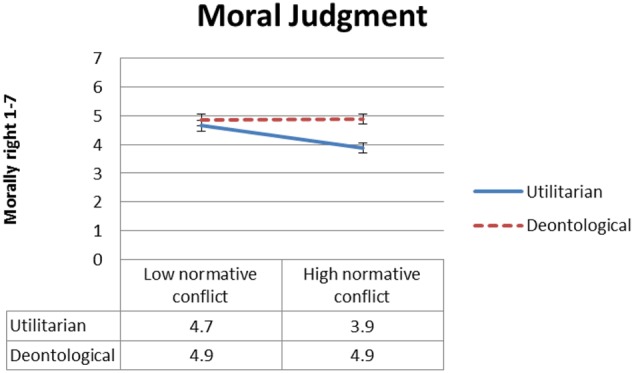
Experiment 1: Mean moral rightness ratings, from 1 (entirely morally wrong) to 7 (entirely morally right), as a function of type of judgment and normative conflict condition. Error bars represent 1 SE. The difference between the low normative conflict condition and the high normative conflict condition is significant for utilitarian (solid blue line) but not for deontological (dashed red line) judgment.

**Figure 2** presents mean ratings of moral rightness as a function of type of moral judgment and goal conflict, and **Figure 3** presents mean ratings of moral rightness as a function of type of moral judgment and normative conflict. To test our *dissociation* and *selective defeasibility* hypotheses, we ran a 2 × 2 × 2 (moral judgment type, normative conflict, and goal conflict) mixed ANOVA. We found the predicted large two-way interaction between type of moral judgment and goal conflict [*F*(1,91) = 10.2, MSE = 1.96, *p* = 0.002, $\eta_{p}^{2}$ = 0.101], with strong effect size. As predicted, moral judgment of the utilitarian action was rated less morally right in the goal suppression condition relative to the no goal suppression condition. Follow-up *t*-tests revealed significant differences in moral rightness rating between the goal suppression condition and the no goal suppression condition for utilitarian moral judgment [*t*(93) = 5.7, *p* < 0.001] but not for deontological moral judgment. The predicted interaction between normative conflict and type of moral judgment was marginal [*F*(1,91) = 3.8, MSE = 1.95, *p* = 0.054, $\eta_{p}^{2}$= 0.040], but follow-up *t*-tests revealed the expected pattern, that is, there were significant differences in moral rightness ratings between the low normative conflict condition and the high normative conflict condition for utilitarian [*t*(93) = 2.90, *p* = 0.005] but not deontological moral judgment.

#### Inference Strength

**FIGURE 4:**
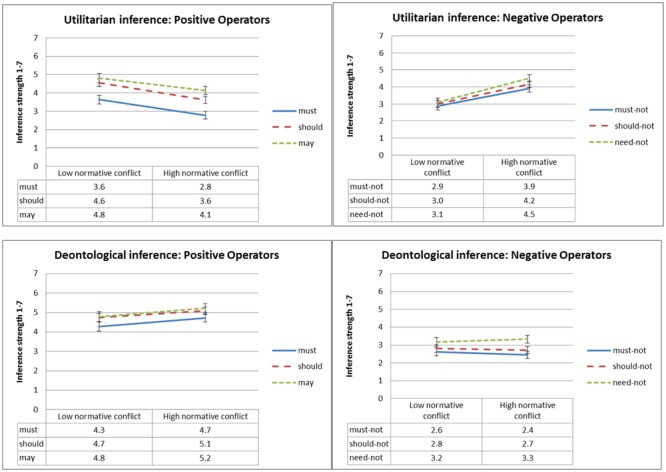
Experiment 1: Mean conclusion ratings, from 1 (Definitely does not follow) to 7 (Definitely follows), as a function of deontic operator, type of inference and normative conflict condition. Error bars represent 1 SE. The difference between the low normative conflict and the high normative conflict condition is significant for all ratings of utilitarian inference strength except ‘may,’ and for none of the ratings of deontological inference strength.

**FIGURE 5:**
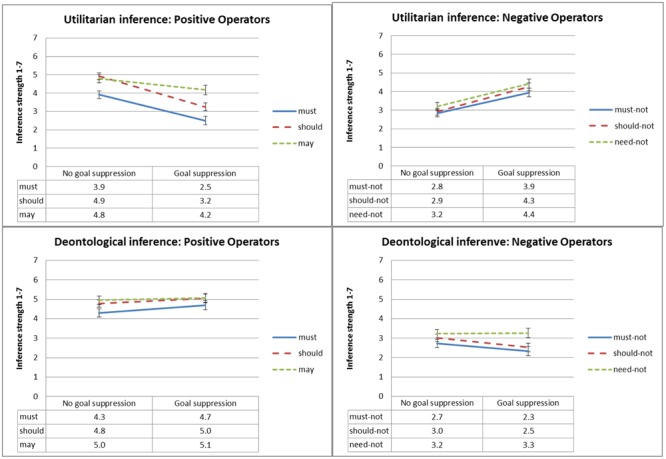
Experiment 1: Mean conclusion ratings, from 1 (Definitely does not follow) to 7 (Definitely follows), as a function of deontic operator, type of inference and goal conflict condition. Error bars represent 1 SE. The difference between the goal suppression and the no goal suppression condition is significant for all ratings of utilitarian inference strength except ‘may,’ and for none of the ratings of deontological inference strength.

**Figure 4** presents mean inference strength ratings as a function of type of inference, deontic operator and normative conflict, and **Figure 5** presents mean inference strength ratings as a function of type of inference, deontic operator and goal conflict. To test our *dissociation* and *selective defeasibility* hypotheses, we ran a 2 × 2 × 2 × 6 (type of inference, normative conflict, goal conflict, and deontic operator) mixed ANOVA. We found the predicted three-way interaction between deontic operator, type of inference, and goal conflict [*F*(2,137) = 16.5, MSE = 6.9, *p* < 0.001, $\eta_{p}^{2}$= 0.153], and the predicted three-way interaction between deontic operator, type of inference, and normative conflict [*F*(2,137) = 10.3, MSE = 6.9, *p* < 0.001,$\eta_{p}^{2}$= 0.101].

As predicted, conclusions with positive modal deontic operators were rated as following more strongly when normative conflict was low relative to the high-conflict condition, and the opposite was true for conclusions with negative modal deontic operators. As illustrated in **Figure 4**, this effect only held for utilitarian inference, whereas deontological inference conclusion ratings were unaffected by normative conflict. Follow-up *t*-tests revealed significant differences in ratings of inference strength between the low- versus high-normative conflict condition for all utilitarian deontic operators [t(93) ≥ 2.7, *p* ≤ 0.008] except ‘may,’ which was marginal given the number of comparisons^2^ [*t*(92) = 2.3, *p* = 0.022]; but no significant differences for the deontological deontic operators.

Similarly and again as predicted, conclusions with positive modal deontic operators were rated as following more strongly under the no goal suppression condition relative to the goal suppression condition whereas the opposite was true for conclusions with negative deontic operators, as can be seen in **Figure 5**. Again this effect only held for utilitarian inference, whereas deontological inference conclusions were not significantly affected by suppression. Follow-up *t*-tests revealed significant differences in inference strength rating between the goal suppression condition and the no goal suppression condition for all utilitarian deontic operators [*t*(93) ≥ 3.3, *p* ≤ 0.001] except ‘may’; and for none of the deontological inference conclusions except ‘should not,’ which was marginal given the number of comparisons [*t*(93) = 2.0, *p* = 0.046]. No other effects were significant.^3^

The results of Experiment 1 support our *dissociation* and *selective defeasibility* hypotheses. In particular, effects of goal conflict were selective in exactly the expected pattern. Utilitarian inference was suppressed when participants were presented with negative consequences of the utilitarian action, with no significant suppression effects on deontological inference. The pattern was the same in the moral judgment tasks: the utilitarian course of action was rated as less morally right when the goal was suppressed, but the deontological course of action was not significantly affected. We found a corresponding pattern with the normative conflict manipulation. Here, too, utilitarian inference, but not deontological inference, was significantly suppressed when participants were presented with a high degree of normative conflict, with a parallel effect in moral rightness, in which the utilitarian course of action was rated as less morally right when the normative conflict was high, but the deontological course of action was not significantly affected.

As an aside, it is worth pointing out that out of the six deontic operators we measured, suppression effects were weakest for the one most closely associated with moral judgment – the deontic operator ‘may,’ which denotes permissibility. Experimental paradigms which focus solely on permissibility as a measure of moral judgment, then, might be in danger of committing a type-II error, as they reject hypotheses that might have been supported had they used other, more sensitive deontic operators.

## Experiment 2

Experiment 1 established the pattern we predicted: utilitarian moral judgment and utilitarian inference were both affected by the same factors, while deontological judgment and deontological inference remained largely unaffected, thus supporting our *dissociation hypothesis* as operationalized in our *selective defeasibility hypothesis.* In Experiment 2, we manipulated a different type of suppression – causality, suppressing an earlier part of the inference chain depicted in **Figure 1**. If the dissociation hypothesis is correct, then weakening the causal link between the action and the outcome should affect utilitarian inference as well as utilitarian moral judgment, but not deontological inference and deontological moral judgment. In other words, we expected to conceptually replicate the results of Experiment 1, but with a very different type of defeater. Such correspondence would lend further support to our *dissociation* and *selective defeasibility* hypotheses.

### Method

#### Participants

A total of 235 participants took part in an online study using the crowd sourcing platform CrowdFlower and were paid a small sum for their participation. All of the participants had a good record of responding appropriately to quality control questions on other CrowdFlower micro jobs. Responses from 47 participants who were non-native speakers of English, dyslexic, failed to sign the informed consent, or failed the validation question were excluded from analysis, resulting in a sample of 188, of which 29 were in the high normative conflict, enhanced causality condition; 34 were in the low normative conflict, enhanced causality condition; 33 were in the high normative conflict, disablers condition; 25 were in the low normative conflict, disablers condition; 34 were in the high normative conflict, alternative causes condition; and 33 were in the low normative conflict, alternative causes condition. As in Experiment 1, there were no significant differences between these six groups in terms of sex, age, or reported level of education.

Also as in Experiment 1, the attrition rate (20%) is comparable to other web studies, and, importantly, there were no significant differences in attrition on the quality control measures between the six experimental test conditions. Participants in the excluded group did not significantly differ from the included group in their age or their reported level of education.

#### Materials and Procedure

The materials and procedure were as in Experiment 1, but without the goal conflict manipulation. This was replaced with a causality manipulation, with three between-participants test conditions: disablers (sufficiency suppression), alternative causes (necessity suppression), and control (enhanced causality). All materials were pretested on a separate sample taken from the same population as the main study (*n* = 71). Participants in the pretest study were asked to rate separately the necessity and sufficiency of the action to the outcome on a 1–7 scale. The items, along with the relevant means of necessity and sufficiency, are reported in Appendix B.

#### Design

As in Experiment 1, except that suppression by goal conflict was replaced with causal suppression, with three levels (enhanced causality vs. disablers vs. alternative causes), manipulated between-participants.

### Results and Discussion

The results broadly replicated those of Experiment 1, with the causal suppression conditions replicating the effects found in the goal suppression conditions.

#### Moral Rightness

**FIGURE 6:**
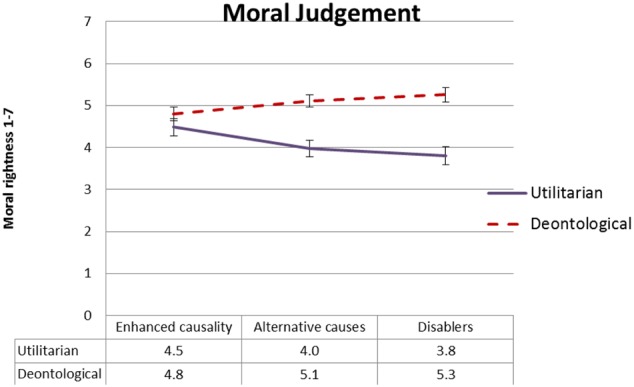
Experiment 2: Mean moral rightness ratings, from 1 (entirely morally wrong) to 7 (entirely morally right), as a function of type of judgment and causal suppression condition. Error bars represent 1 SE.

**FIGURE 7:**
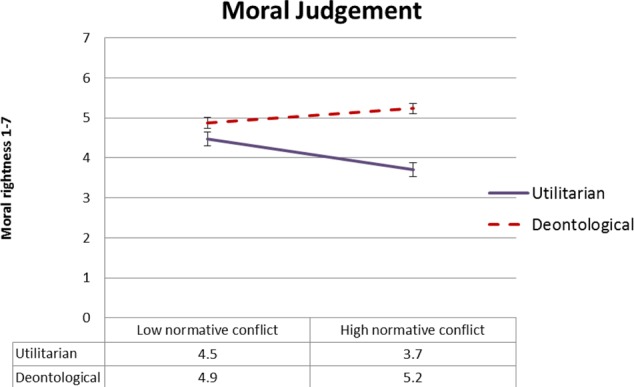
Experiment 2: Mean moral rightness ratings, from 1 (entirely morally wrong) to 7 (entirely morally right), as a function of type of judgment and normative conflict condition. Error bars represent 1 SE.

**Figure 6** presents mean ratings of moral rightness as a function of type of moral judgment and causal suppression, and **Figure 7** presents mean ratings of moral rightness as a function of moral judgment type and normative conflict. To test our *dissociation* and *selective defeasibility* hypotheses, we ran a 2 × 2 × 3 (moral judgment type, normative conflict, and causal suppression) mixed ANOVA. We found the predicted two-way interaction between type of moral judgment and causal suppression [*F*(2,154) = 3.78, MSE = 2.29, *p* = 0.025, $\eta_{p}^{2}$ = 0.047], with moderate effect size. As predicted, the utilitarian action was rated less morally right in the causal suppression conditions relative to enhanced causality condition. Planned comparisons contrasting the enhanced causality condition with each of the causal suppression conditions, respectively, revealed significant differences in moral rightness rating between the enhanced causality condition and the disabler condition for utilitarian moral judgment [*t*(159) = 2.4, *p* = 0.017], although the difference between the enhanced causality condition and the alternative causes condition was borderline given the number of comparisons [*t*(159) = 2.1, *p* = 0.038]. Ratings of moral rightness of the deontological action did not differ significantly between the enhanced causality condition and the causal suppression conditions (*p* ≥ 0.06).

We also found a two-way interaction between type of moral judgment and normative conflict [*F*(1,154) = 10.8, MSE = 2.29, *p* = .001, $\eta_{p}^{2}$ = 0.065], in which judgment of moral rightness of the utilitarian action was higher in the low normative conflict condition, and the opposite was true of the moral rightness of the deontological action. Follow-up *t*-tests revealed both effects to be significant [*t*(159) = 3.5, *p* = 0.001; and *t*(160) = 2.3, *p* = 0.025, respectively].

#### Inference Strength

**FIGURE 8:**
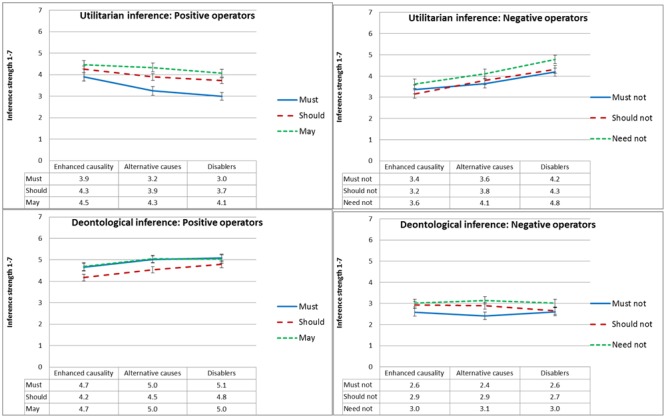
Experiment 2: Mean conclusion ratings, from 1 (Definitely does not follow) to 7 (Definitely follows), as a function of deontic operator, type of inference and causal suppression condition. Error bars represent 1 SE.

**FIGURE 9:**
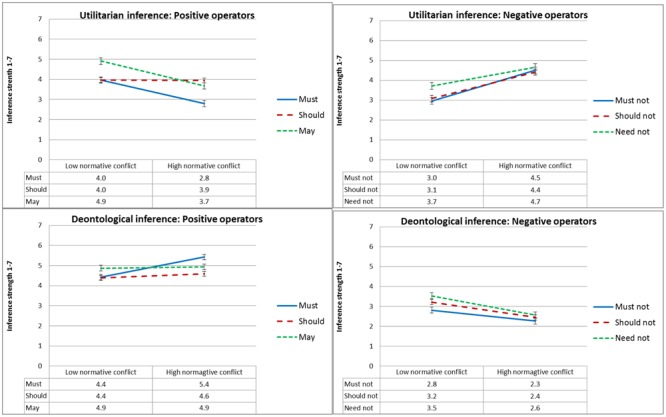
Experiment 2: Mean conclusion ratings, from 1 (Definitely does not follow) to 7 (Definitely follows), as a function of deontic operator, type of inference and normative conflict condition. Error bars represent 1 SE.

**Figure 8** presents mean ratings of inference strength as a function of type of inference, deontic operator and causal suppression, and **Figure 9** presents mean ratings of inference strength as a function of type of inference, deontic operator and normative conflict. To test our *dissociation* and *defeasibility* hypotheses, we ran a 2 × 2 × 3 × 6 (type of inference, normative conflict, causal suppression, and deontic operator) mixed ANOVA. We found the predicted three-way interaction between deontic operator, type of inference, and causal suppression [*F*(4,278) = 5.1, MSE = 1.9, *p* = 0.001, $\eta_{p}^{2}$ = 0.062], as well as a three-way interaction between deontic operator, type of inference, and normative conflict [*F*(2,277) = 37.0, MSE = 1.9, *p* < 0.001, $\eta_{p}^{2}$= 0.195].

As expected, conclusions with positive modal deontic operators were rated as less strongly following when causality was suppressed by alternative causes or disablers relative to the enhanced causality condition, and the opposite was true for conclusions with negative deontic operators, but, as illustrated in **Figure 8**, this effect only held for utilitarian inference, whereas deontological inference conclusions were not significantly affected. Planned contrasts revealed, for utilitarian inference, significant differences in ratings of inference strength between the enhanced causality condition and the disablers condition for the deontic operators ‘must,’ ‘must not,’ ‘should-not,’ and ‘need-not’ [*t*(158) ≥ 2.7, *p* ≤ 0.008], and marginal differences (given the number of comparisons) for the deontic operator ‘should’ [*t*(158) = 2.5, *p* = 0.013]. In contrast, deontological inference ratings were largely unaffected, with no significant differences for five out of six deontic operators. The two exceptions were ‘should’ [*t*(158) = 2.9, *p* = 0.004], and ‘must,’ with a marginal difference given the number of comparisons [*t*(158) = 2.0, *p* = 0.049].

The effect was similar, although slightly less robust, for the differences between the enhanced causality condition and the alternative causes condition. For utilitarian inference, planned contrasts revealed marginal differences, given the number of comparisons, in ratings of inference strength between the enhanced causality condition and the alternative causes condition for the deontic operators ‘must’ [*t*(158) = 2.4, *p* = 0.017] and ‘should not’ [*t*(158) = 2.4, *p* = 0.020]. Deontological inference ratings were unaffected, with no significant or even marginal differences for any of the six deontic operators.

We also found a three-way interaction between normative conflict, deontic operator, and type of inference, illustrated in **Figure 9**, although it took a somewhat different form than expected. For utilitarian inference, we replicated the results of Experiment 1, with inference strength ratings of positive operators going up in the low normative conflict condition, and inference strength ratings of negative operators going down. However, unlike Experiment 1, in which deontological inference was unaffected, in this experiment deontological inference was affected in the opposite direction to that of utilitarian inference, with inference strength ratings of negative operators going up in the low normative conflict condition, and inference strength ratings of the positive operator ‘must’ going down in the low normative conflict condition. Follow-up *t*-tests revealed significant differences between the high normative conflict and the low normative conflict condition for all deontic operators in both types of inference [*t*(158) ≥ 3.8, *p* < 0.001]. The exceptions were utilitarian and deontological inference conclusions with ‘should,’ and deontological inference conclusions with ‘may,’ in which differences were not significant; and the deontological inference with ‘must not’ which was marginal given the number of comparisons [*t*(158) = 2.7, *p* = 0.009].^4^

Our manipulation of causal strength provided solid support for our *dissociation* and *selective defeasibility* hypotheses. The effects of causal suppression, and those of sufficiency suppression in particular, replicated the effects of goal suppression from Experiment 1 with a very similar level of selectivity. Utilitarian inference, but not deontological inference, was attenuated when sufficiency (and to some extent, necessity) was suppressed, with a parallel effect on moral rightness, in which the utilitarian course of action was rated as less morally right when the goal was suppressed, but the deontological course of action was not significantly affected.

For utilitarian judgments, the normative conflict manipulation provided a clear replication of Experiment 1, in which conclusions with positive deontic operators were rated as stronger in the low-conflict relative to the high-conflict condition. Unexpectedly, it also affected deontological judgment, albeit in the opposite way. Our interpretation of these findings is that conflict manipulation, by definition, creates an explicit competition between the utilitarian course of action and the deontological one. One can take either the utilitarian action (e.g., get the sect members to eat more fruit and vegetables), or the deontological action (e.g., respect the sect members’ religious beliefs). It is impossible to do both. Conflict focuses attention on the contrast between the deontological and utilitarian actions, and so may increase people’s sensitivity to it. Participants might have become more aware of the conflict in Experiment 2 relative to Experiment 1 due to the way that the causality manipulation in Experiment 2 highlighting the causal path of the recipient rather than that of the actor, a manipulation known to strengthen deontological responding (Waldmann and Dieterich, 2007).

## General Discussion

Faced with a description of an action with a good or bad outcome, people readily infer normative (‘deontic’) conclusions. Thus, if fruit and vegetables are what *will* save the sect members from scurvy, then Ben *should* get them to eat more fruit and vegetables. The ‘will’ is descriptive; the ‘should’ is normative, or in logical terms, *deontic*. This type of inference (dubbed ‘deontic introduction’ in previous work) allows humans to generate novel normative and moral rules where none existed before. In this paper, we set out to explore the role of deontic introduction in moral judgment, and in particular the type of moral judgment that relies on calculating the costs and benefits of the consequences of one’s actions – utilitarian, or consequentialist moral judgment. We expected deontic introduction to cohere exclusively with utilitarian but not with deontological moral judgment. Thus, the moral rightness of Ben’s getting the sect members to eat more fruit and vegetables is associated with the descriptive-to-normative inference that this is what Ben should (must and may) do. In contrast, the moral rightness of Ben respecting the sect members’ religious beliefs (hence *not* getting them to eat more fruit and vegetables) is relatively independent of such inference.

In two experiments, we used a range of suppression manipulations, including causal suppression, goal conflict, and normative conflict. Across these disparate manipulations, we found very similar patterns of selective defeasibility. When utilitarian inference (which measured deontic introduction) was suppressed, so was the perceived moral rightness of the utilitarian course of action. If a normative conflict led people to believe that getting the sect members to eat more fruit and vegetables would be offensive to their beliefs, they stopped inferring that Ben should get the sect members to eat more fruit and vegetables, and in parallel, they also thought such action less morally right. A similar thing happened when goal conflict led participants to believe that getting the sect members to eat more fruit and vegetables would result in a bad outcome (massive allergic reaction); and again when causal suppression led participants to believe that eating more fruit and vegetables was insufficient for overcoming scurvy (because the sect’s staple food blocked vitamin C from absorbing); or that eating fruit and vegetables was unnecessary (because they could get vitamin C from animal liver). In most cases this suppression pattern was highly selective: inference strength and moral rightness of the utilitarian action were significantly affected, whereas inference strength and moral rightness of the deontological action were much less affected, or, in one case, affected in the opposite direction. The consistency of the selective pattern across so many manipulations is at least suggestive. Thus, we have a good level of support for our dissociation hypothesis, that deontic introduction *exclusively coheres* with utilitarian moral judgment – that is, that deontic introduction is associated with utilitarian moral judgment but dissociated with deontological moral judgment.

It is noteworthy that the selective suppression pattern occurred even though, by the very nature of moral dilemmas, the two courses of action are mutually and logically contradictory. Thus, if one *must* respect the religious beliefs of the sect members, then one *must not* try to get them to eat more fruit and vegetables, and vice versa; and if it is *permissible* to get them to eat more fruit and vegetables, then it is *impermissible* to respect their religious beliefs. We seem to have unearthed a focusing bias, or framing effect, peculiar to moral dilemmas, that we would never have found with the usual trolley paradigm. In the classic framing paradigm (Tversky and Kahneman, 1981) people become asymmetrically risk-averse versus risk-seeking, depending on whether the problem is depicted in terms of gains or losses, respectively; here, people become asymmetrically action-averse depending on whether the problem is depicted in utilitarian or deontological terms.

### Holyoak and Powell’s Deontological Coherence Framework

We noted earlier that no other theory predicts quite the pattern that we predicted, and supported. One prominent theory which differs in its predictions is the recent major theoretical framework, *deontological coherence* (Holyoak and Powell, 2016). Although there are some interesting parallels between the deontological coherence framework and our theory, there are crucial differences as well, as we now show.

The deontological coherence framework proposes that moral judgment typically involves multiple factors, some deontological, some consequential/utilitarian, as well as causal knowledge, theory of mind, and emotional factors. The result is an emergent ‘local coherence’ between the outcome of the moral judgment and the contributing factors. This often leads to what Holyoak and Powell call ‘bidirectional inferences,’ in which beliefs, intention attribution and emotions are revised post-judgment to become more coherent with whatever moral judgment was passed. This part of the deontological coherence framework fits well with the defeasible nature of deontic introduction, and indeed our own findings show clear support for bidirectional inference: people indeed revised their moral judgment when a range of defeaters suppresses their utilitarian inference.

The deontological coherence framework integrates Cheng and Holyoak’s (1985) theory of pragmatic reasoning schemas, an early theory developed to account for reasoning on the Wason selection task (Wason, 1966). In the standard version of this task, participants are presented with a conditional rule of the form *if p, then q*, e.g., ‘if there is an A on one side of the card, then there is a 6 on the other side’; and a set of four cards, each with a letter on one side and a digit on the other side. The exposed values on the cards match the values of *p, not-p, q*, and *not-q*, respectively; for example, A, F, 6, and 4. Participants are instructed to turn over the cards – and only the cards – that would allow them to test if the rule is true or false in respect to the cards. Only a small minority of participants, typically around 10%, select the classically normative combination, *p* and *not-q* (A and 4, respectively, in our example). However, this dramatically changes when the rule is deontic (as Cheng and Holyoak were the first to identify). For example, in Griggs and Cox’s (1982) drinking age rule, participants are invited to imagine that they are a police officer tasked with enforcing a rule ‘If a person drinks beer, then this person must be over 21 years of age,’ and the cards (each of which represents a drinker at a bar) are *beer, coke, 22*, and *16*, respectively. When participants are instructed to turn over the cards that would allow them to tell of the rule was being violated, performance improves substantially, with about 75% of participants choosing the normative *beer* and *16* cards (see Evans and Over, 2004, Chapter 5, for a review).

Cheng and Holyoak’s (1985) theory explained this facilitation effect by proposing pragmatic reasoning schemas, a set of generalized, context-sensitive rules defined in terms of goals and relationship to these goals. Obligations, permissions, and causations are all types of pragmatic reasoning schemas. A later development (Holyoak and Cheng, 1995) formalized such schemas in terms of rights and duties – that is, purely deontological terms. This is the interpretation that Holyoak and Powell (2016) draw on to integrate into their theory, arguing that this suggests that people draw on intuitive deontological concepts of rights and duties to understand social regulations.

We note, however, that other major theoretical developments in the field of reasoning have provided a utilitarian rather than deontological interpretation of the literature on the deontic selection task. For example, Manktelow and Over (1991) provided a utilitarian interpretation of perspective effects in the deontic selection task, demonstrating that different utilities ties with various social roles drive different patterns of card selection. Similarly, Oaksford and Chater (1994) suggested a Bayesian framework for the selection task, which also relies on analysis of utilities.

Nevertheless, we find much to admire in the deontological coherence framework. One very important feature relates to the *methodological atheism* which underlies the deontological coherence framework. Holyoak and Powel argue that, since there is no widely accepted normative standard for moral judgment, attempts to regard as psychologically inferior any moral judgment which is not strictly utilitarian/consequentialist are ill-advised. Thus, they reject Sunstein’s (2005) moral heuristics approach, which regards departure from utilitarian rules as biased; and they object to Greene’s dual processing theory, partly on the grounds that Greene sees deontological judgment as ethically inferior (Greene, 2008).

The stance of methodological atheism is very close to the *descriptivist* position advocated by two of the authors of the current manuscript (Elqayam and Evans, 2011; Evans and Elqayam, 2011). Elqayam and Evans argued that any normative evaluations are counterproductive in the psychological study of reasoning and decision making. The business of psychological science is to describe behavior rather than evaluate or dictate it. Not only is there a lack of consensus on normative standards in the psychology of reasoning and decision making; any attempt to resolve this by drawing on empirical evidence is in itself non-normative, drawing as it does on an *is-to-ought* inference (see previous section, “How Do Descriptions Become Norms?). It is easy to see how descriptivism resonates with methodological atheism. The objections which Elqayam and Evans raise are doubly true for the study of moral judgment, where the lack of consensus is even more pronounced. More generally, Holyoak and Powell object to Greene’s dual process theory because it regards deontological processes as type 1 processes. We certainly concur with this: as commented in the “Introduction,” the evidence for dual processing in moral judgment is equivocal. We remain agnostic on whether people have a single unified system for moral judgment as Holyoak and Powell suggest, or dual systems.

However, there is also a clear point of disagreement between our theory and the deontological coherence framework. Within the latter, all elements in moral judgment interact; importantly, Holyoak and Powell reject a possibility of dissociation between these elements, proposing that ‘the inputs to moral judgment processes are intertwined *rather than dissociated’* (p. 1193, italics ours). This is where we differ: we contest the deontological coherence framework’s claim that such factors are intertwined rather than dissociated. Such claim seems to us premature. Instead, we suggest that a more productive research question for the deontological coherence framework would focus on the boundaries of this principle: when do factors affecting moral judgment dissociate, and when do they intertwine?

Our findings show that, while utilitarian judgments are indeed affected by utilitarian, causal, and deontological factors, this is not the case for deontological judgments, which are much less sensitive to utilitarian and causal factors. Thus, ratings of the morality of the utilitarian option were affected by normative conflict (deontological), goal conflict (utilitarian), and causal suppression (causal); while ratings of the morality of the deontological option remained largely unaffected. While the former is in accord with the deontological coherence framework, the latter directly contradicts it. We do not claim that our findings are in any way exhaustive: it is entirely conceivable that deontological moral judgment might be affected by some of these factors in a different type of context, or by different factors. We identify this as an essential question for future research. An important caveat is that any such mapping cannot draw on the typical trolley-type dilemmas, since their forced-choice paradigm risks creating an artifact. Any experimental design looking for dissociations should examine utilitarian and deontological actions separately, as we have.

### Final Thoughts

The pattern we found suggests a family of informal inferences, of which deontic introduction and utilitarian moral judgment are members; but not deontological inference and deontological moral judgment. What these types of inference have in common is that they are *norm-generating, consequential*, and *utilitarian*: they draw novel, tailor-made normative conclusions from descriptive premises, based on evaluation of how good or bad the consequences might be. Deontic introduction and utilitarian moral judgment share these features. Deontological inference and deontological moral judgment do not: they reflect pre-existing norms instead. Hence, what affects deontic introduction affects perception of utilitarian moral rightness, selectively and exclusively. In contrast, no variables selectively affected ratings deontological moral rightness. Admittedly, the selective defeasibility pattern was not entirely pure. Still, utilitarian moral judgment patterns are remarkably similar to those of deontic introduction. We should also emphasize that our position is strictly that of methodological atheism: this is a psychological observation and not an ethical one. In no way does it reflect on the moral worthiness of either deontological or utilitarian judgment. (Indeed, to draw an ethical conclusion here would be to commit an ‘is-ought’ fallacy.)

This family of norm-generating inferences we suggest includes more than deontic introduction and utilitarian moral judgment. There are other members, mostly other forms of informal argumentation. Slippery slope arguments are a good example (Corner et al., 2011): in this type of informal argumentation, people draw negative normative conclusions from negative descriptive premises, such as “legalization of euthanasia will lead to an increase in the number of instances of ‘medical murder”’, with the invited conclusion that ‘euthanasia should not be legalized.’ Similarly, persuasions and dissuasions (Thompson et al., 2005) are informal arguments expressed as conditional sentences with negative or positive outcome, such as ‘If Britain leaves the European Union, there will be a severe recession.’ This invites a matching normative conclusion – in this case, it invites the listener to infer that Britain should not leave the European Union. Conceivably, deontic introduction is necessary to all these inferences.

The novelty of our contribution is twofold. While utilitarian moral judgment is by definition consequential and utilitarian, little attention has been hitherto paid to its inferential and norm-generating characteristics. Both aspects are worthy of theoretical attention. That we can generate novel norms allows us to create solutions to novel situations and direct future action, a feature which takes on particular significance in the context of novel moral dilemmas. The way that utilitarian judgment allows us to create new normative rules adapted to a changing world is by drawing deontic introduction inference.

The second advantage of this novel conceptual framework is that it integrates moral judgment research back into the study of thinking and reasoning, with corresponding theoretical and practical benefits to be reaped (Bonnefon, 2013). Recent work in the field of moral judgment tends to prefer the term ‘moral judgment’ to ‘moral reasoning,’ perhaps because the latter had become associated with the strongly Kantian approach of Piaget (1997) and Kohlberg (Kohlberg and Hersh, 1977). This, however, does not and should not preclude discussion of the *inferential* aspects of moral judgment. Such integration is fully within the spirit of the new paradigm in psychology of reasoning (Over, 2009; Oaksford and Chater, 2009; Elqayam and Over, 2013). The new paradigm in psychology of reasoning is a decision-theoretic, Bayesian approach to reasoning. It regards reasoning as a branch of decision making, sensitive to the same parameters. Reasoning, according to the new paradigm, is based on degrees of belief rather than black-and-white, binary truth values; it is defeasible; and it is sensitive to what we desire and do not desire – utility, in other words. Our family of norm-generating inferences is very much a new paradigm family, containing as it does a host of informal inferences, and regarding moral judgment as a type of informal inference.

We also see benefit in designing new experimental paradigms for moral judgment research that would liberate the field from its dependence on forced-choice trolley problems. The controversy around dual processing in moral judgment seems to have reached a standstill; more sensitive measures could be useful for advancing the debate.

Research in moral judgment has made progress in leaps and bounds since the turn of the century. Yet much of it remained curiously isolated from relevant work within the field of reasoning, especially work on deontic reasoning. A closer integration can only benefit both fields. In this paper we have made a crucial step toward such integration.

## Ethics Statement

De Montfort University, Faculty of Health and Life Sciences, Faculty Research Ethics Committee. Participants were presented with an information sheet and an informed consent form. No vulnerable participants were involved.

## Author Contributions

SE: Leading on theoretical development, design of studies, and write-up of publication. MR: Contributor to data collection, data analysis, theoretical development, design of studies, and write-up of publication. VT, DO, and JE: Contributor to theoretical development, design of studies, and write-up of publication.

## Conflict of Interest Statement

The authors declare that the research was conducted in the absence of any commercial or financial relationships that could be construed as a potential conflict of interest.

## Footnotes

1 Taken from Pennycook et al. (2014). At the end of the demographics section, participants were given a list of hobbies and instructed: “If you are reading these instructions please write ‘I read the instructions’ in the ‘other’ box.”

2 Bonferroni 0.05/6 = 0.008.

3 Following a reviewer’s suggestion, we collapsed all positive operators versus all negative operators and ran an additional 2 × 2 ANOVA with type of operator (positive vs. negative) and normative conflict condition (high vs. low) as the within- and between-participants independent variables, respectively, and inference strength as the dependent variable. The operator × normative conflict interaction was not significant, *F*(1,93) = 1.3, *p* = 0.25; contrast estimate 0.468; 95% CI [-0.336, 1.272].

4 Following a reviewer’s suggestion, we collapsed all positive operators versus all negative operators and ran an additional 2 × 2 ANOVA with type of operator (positive vs. negative) and normative conflict condition (high vs. low) as the within- and between-participants independent variables, respectively, and inference strength as the dependent variable. The operator × normative conflict interaction was significant, *F*(1,160) = 23.5, *p* < 0.001, $\eta_{p}^{2}$ine-formula>= 0.128; contrast estimate 1.22, 95% CI [-1.71, -0.72].
