# Supplementary material for: Utilitarian Moral Judgment Exclusively Coheres with Inference from Is to Ought
Source: Front Psychol. 2017 Jun 22;8:1042. doi: 10.3389/fpsyg.2017.01042 (PMC5480028; doi:10.3389/fpsyg.2017.01042)
Supplement: Supplementary file 1 [file Data_Sheet_1.pdf]

Appendices for:

Elqayam, S., Wilkinson, M. R., Thompson, V. A., Over, D. E., & Evans, J. S. B. T., (2017). Utilitarian moral judgment exclusively coheres with inference from is to ought. *Frontiers in Psychology: Cognitive Science, 8,* 1042. doi: 10.3389/fpsyg.2017.01042

Appendix A: Items in Experiment 1

Vitamin C

Ben is an anthropologist working with a religious sect in rural America. He gradually realises that many of his informants suffer from joint pains, bleeding gums, and they bruise easily. He discovers that the sect's religious beliefs severely limit their fruit and vegetables intake. The result is that many of them suffer from debilitating vitamin C deficiency (scurvy). Ben considers persuading them to eat more fruit and vegetables.

High normative conflict: However, this would be against their religion. The code of practice of the Anthropological Association requires anthropologists to respect the beliefs of their informants.

If Ben gets the sect members to eat more fruit and vegetables, he will save them from vitamin C deficiency.

Goal suppression: However, the sect members suffer from a rare form of allergy to fruit and vegetables. If Ben gets the sect members to eat more fruit and vegetables, it will drive them into severe, life-threatening allergic reaction.

Charity

Sam has worked for many years in the pharmaceutical industry and now heads a charity which supports patients with a rare neurological disease. The patients are in need of extended care and the charity is struggling to provide help. A pharmaceutical company which develops a medicine for this disease has offered substantial financial support to the charity, on the condition that Sam will recommend the medicine to the patients. The medicine is effective in alleviating the worst symptoms of the disease.

High normative conflict: The charities' code of practice requires workers to keep impartiality, and so forbids recommending a particular medicinal product.

If Sam recommends the medicine, the charity will provide support for many more patients with the rare disease.

Goal suppression: However, the charity is also supported by a rival pharmaceutical firm. If Ben recommends the medicine, the rival firm will withdraw its support, resulting in a large loss to the charity.

Pseudonyms

Stella is an anthropologist working in a small village in South America. She has published a best-selling book with a detailed account of the village life, and is now working on a second book about the same village. When Stella returns to the village, she gives the first book as a present to the village elders. They are pleased with her report, but puzzled that she 'got their names wrong', as she used pseudonyms.

High normative conflict: Stella had followed the code of practice of the Anthropological Association, replacing real names with pseudonyms, to protect informants from possible harm.

The villagers now ask her to disclose the real names of the village and the people in the second book, so that they can use the book as evidence to apply for UNICEF support to build a computer lab for the local school.

If Stella discloses real names in her second book about the village, the village will receive financial support to build a computer lab for the local school.

Goal suppression: However, some people in the village are under a witness protection scheme, having testified against a powerful mafia leader. If Stella discloses real names in her second book about the village, it will expose them to risk.

Appendix B: Items in Experiment 2 (with necessity and sufficiency ratings)

Mean sufficiency and necessity ratings are from the pretest.

Control n=24; sufficiency suppression n = 24; necessity suppression n = 23

Vitamin C

Ben is an anthropologist working with a religious sect in rural America. He gradually realises that many of his informants suffer from joint pains, bleeding gums, and they bruise easily. He discovers that the sect's religious beliefs severely limit their fruit and vegetables intake. The result is that many of them suffer from debilitating vitamin C deficiency (scurvy). Ben considers persuading them to eat more fruit and vegetables.

High normative conflict: However, this would be against their religion. The code of practice of the Anthropological Association requires anthropologists to respect the beliefs of their informants.

Causal enhancement: The sect members also avoid any medication or food supplements. The only way to save them from vitamin C deficiency is to get them to eat more fruit and vegetables. Research shows that people with vitamin C deficiency only need to eat fruit and vegetables in order to recover very quickly. [Sufficiency M=6.0; necessity M=5.3.]

If Ben gets the sect members to eat more fruit and vegetables, he will save them from vitamin C deficiency.

Sufficiency suppression (disabler): However, it is not enough for Ben to get the sect members to eat more fruit and vegetables. Their staple food is a local type of shellfish, which contains a substance that does not allow the body to absorb vitamin C at all. [Sufficiency M=2.9]

Necessity suppression (alternative cause): However, it is not necessary for Ben to get the sect members to eat more fruit and vegetables. He can also advise them to eat more beef liver, which contains plenty of vitamin C, and is not against the sect's beliefs. [Necessity M=3.1]

Charity

Sam has worked for many years in the pharmaceutical industry and now heads a charity which supports patients with a rare neurological disease. The patients are in need of extended care and the charity is struggling to provide help. A pharmaceutical company which develops a medicine for this disease has offered substantial financial support to the charity, on the condition that Sam will recommend the medicine to the patients. The medicine is effective in alleviating the worst symptoms of the disease.

High normative conflict: The charities code of practice requires workers to keep impartiality, and so forbids recommending a particular medicinal product.

Causal enhancement: Sam has already tried applying to government funds, philanthropic institutes and private donors, but funding was meagre and the charity is still struggling. The only way to support more patients is to recommend the medicine. Sam only needs to recommend the medicine in order to have sufficient funds from the pharmaceutical company to help the patients. [Sufficiency M=5.8; necessity M=5.3]

If Sam recommends the medicine, the charity will provide support for many more patients with the rare disease.

Sufficiency suppression (disabler): However, it is not enough for Sam to recommend the medicine. The pharmaceutical company has recently gone bankrupt and is unable to provide any financial support. [Sufficiency M=3.1]

Necessity suppression (alternative cause): However, it is not necessary for Sam to recommend the medicine. The charity is eligible to receive governmental financial support which is available to all charities working with neurological illness. [Necessity M=3.5]

Pseudonyms

Stella is an anthropologist working in a small village in South America. She has published a best-selling book with a detailed account of the village life, and is now working on a second book about the same village. When Stella returns to the village, she gives the first book as a present to the village elders. They are pleased with her report, but puzzled that she ‘got their names wrong’, as she used pseudonyms.

High normative conflict: Stella had followed the code of practice of the Anthropological Association, replacing real names with pseudonyms, to protect informants from possible harm.

The villagers now ask her to disclose the real names of the village and the people in the second book, so that they can use the book as evidence to apply for UNICEF support to build a computer lab for the local school.

Causal enhancement: The villagers have already tried applying for funding several times, but all their applications were rejected due to lack of evidence from reliable external sources. The only way for the village to receive financial support for their computer lab is by being named in Stella's book so they can submit it as reliable evidence in their application. They have a very strong case, and only need this piece of evidence to secure the funding. [Sufficiency M=5.0; necessity M=5.4]

If Stella discloses real names in her second book about the village, the village will receive financial support to build a computer lab for the local school.

Sufficiency suppression (diabler): However, it is not enough for Stella to disclose real names in her second book about the village. The village is not eligible to apply for UNICEF funding. [Sufficiency M=3.8]

Necessity suppression (alternative cause): However, it is not necessary for Stella to disclose real names in her second book about the village. The village has just received a large grant from a charity supporting education in rural South America. [Necessity M=3.1]
